# The crosstalk between reactive oxygen species and noncoding RNAs: from cancer code to drug role

**DOI:** 10.1186/s12943-021-01488-3

**Published:** 2022-01-26

**Authors:** Jing Zuo, Zhe Zhang, Maomao Li, Yun Yang, Bohao Zheng, Ping Wang, Canhua Huang, Shengtao Zhou

**Affiliations:** 1 State Key Laboratory of Biotherapy and Cancer Center, West China Hospital, and West China School of Basic Medical Sciences & Forensic Medicine, Sichuan University, and Collaborative Innovation Center for Biotherapy, Chengdu, 610041 People’s Republic of China; 2 Department of Obstetrics and Gynecology, Key Laboratory of Birth Defects and Related Diseases of Women and Children of MOE and State Key Laboratory of Biotherapy, West China Second University Hospital, Sichuan University and Collaborative Innovation Center, Chengdu, People’s Republic of China

**Keywords:** Reactive oxygen species, Oxidative stress, Noncoding RNAs, Cancer diagnosis, Cancer therapy

## Abstract

Oxidative stress (OS), characterized by the excessive accumulation of reactive oxygen species (ROS), is an emerging hallmark of cancer. Tumorigenesis and development driven by ROS require an aberrant redox homeostasis, that activates onco-signaling and avoids ROS-induced programmed death by orchestrating antioxidant systems. These processes are revealed to closely associate with noncoding RNAs (ncRNAs). On the basis of the available evidence, ncRNAs have been widely identified as multifarious modulators with the involvement of several key redox sensing pathways, such as NF-κB and Nrf2 signaling, therefore potentially becoming effective targets for cancer therapy. Furthermore, the vast majority of ncRNAs with property of easy detected in fluid samples (e.g., blood and urine) facilitate clinicians to monitor redox homeostasis, indicating a novel method for cancer diagnosis. Herein, focusing on carcinoma initiation, metastasis and chemoradiotherapy resistance, we aimed to discuss the ncRNAs-ROS network involved in cancer progression, and the potential clinical application as biomarkers and therapeutic targets.

## Introduction

Intracellular oxidative reactions play a vital role in metabolic processes, by which plenty of biomolecular compounds like reactive species are produced. Under normal physiological conditions, these products maintain proper concentrations, with a close relation to enzyme activation [1–3], protein synthesis [4, 5], signal transduction [6, 7] and gene expression [8, 9]. However, excessive products have some detrimental impacts, probably leading to the dysregulation of the above biological events and causing all different kinds of diseases, including inflammation [10], diabetes [11], cardiovascular disease [12] and even malignancies [13]. Therefore, the maintenance of redox homeostasis is essential to normal cellular activities and human health. There are four main reactive species: reactive oxygen species (ROS), reactive nitrogen species (RNS), reactive sulfur species (RSS) and reactive chlorine species (RCS). ROS is the most abundantly produced and frequently explored, involving superoxide anion (O2−), hydrogen peroxide (H_2_O_2_), hydroxyl radical (OH−), singlet oxygen (1O2) and ozone (O3) [14]. ROS originates from internal oxygen metabolism and external environmental changes. Internal sources stem from oxygen, which is catalyzed by different enzymes, and primarily occurs in mitochondria, peroxisomes and endoplasmic reticulum (ER) [15] . Meanwhile, external inducers are composed of ultraviolet radiation, ionizing radiation and toxic compounds, which generate accumulated ROS and probably cause cancerous transformation [16–18].

OS refers to the equilibrium disturbance of oxidative and anti-oxidative systems in favor of oxidant burden, which is generally mediated by ROS. Normally, intracellular antioxidants are enough to neutralize extra oxides to maintain homeostasis and ensure a balanced status. However, when redox balance is broken, extra ROS will induce OS and damage biomacromolecules, including DNA [19–21], RNA [22, 23], proteins [24] and lipids [25–27], engendering cell death or provoking the malignant transformation of normal cells. Intriguingly, excessive ROS seems to preferentially accumulate in cancer cells. Thus, there are two interesting issues worthy of discussion: how cancer cells protect themselves from ROS toxicity and whether ROS-based anticancer strategies benefit cancer therapy.

NcRNAs are defined as transcripts that cannot be translated into proteins or functional proteins at least. In recent years, ncRNAs have attracted much interest and been proved to be direct or indirect elements of gene transcriptional and post-transcriptional regulation [28–31]. A large number of ncRNAs have been identified, which are classified into two major groups in length: short ncRNAs (< 200 bp) and long ncRNAs (lncRNAs) (> 200 bp). Short ncRNAs mainly comprise microRNAs (miRNAs), circular RNAs (circRNAs), small interfering RNAs (siRNAs), small nuclear RNAs (snRNAs), small nucleolar RNAs (snoRNAs), PIWI-interacting RNAs (piRNAs), tRNA-derived small RNAs (tsRNAs) and enhancer noncoding RNAs (eRNAs) [32, 33]. A growing number of ncRNAs are regarded as possible diagnostic and prognostic biomarkers of cancer dynamics and treatment monitoring, for instance, circulating U2 small nuclear RNA functioned as a biomarker in epithelial ovarian cancer and lymphoma [34, 35]. Furthermore, considering the widespread functions of ncRNAs in cancer, the enormous therapeutic potential of ncRNAs is nonnegligible. siRNAs possess the advantages of smaller size, targetable features and simplex working principle, making it possible to treat cancer by silencing key oncogenes with the extensive application of nanotechnology [36, 37]. Additionally, the interplay of ncRNAs with ROS has also been proved extensively, thereby a considerable treatment method referring to ncRNAs could be built on the basis of ROS. CircRNA-101,036, identified as a tumor suppressor, represses cancer development by inducing ER stress and substantial ROS accumulation in oral squamous cell carcinoma [38]. These results reveal that ncRNAs can become promising therapeutic targets and agents based on ROS.

For many years, most studies have been addicted to the drivers of cancer initiation and progression, such as aberrant expression of oncogenes and tumor suppressors or dysregulation of pivotal signaling pathways. They are ever considered as promising targets for cancer therapy, and some pharmaceutical companies have developed drugs based on these targets. Nevertheless, severe side-effects, low sensitivity and therapeutic resistance greatly limit their further application. Cancer cells have many unique characteristics compared with normal cells, including immortalization, metastasis, aerobic glycolysis, gene mutation and immune evasion. Targeting these characteristics may provide more optional treatment methods to kill cancer cells while sparing normal cells. OS is one of the most significant hallmarks within cancer cells, and has been reported to influence ncRNAs. NcRNAs can also modulate ROS in many ways, so profoundly understanding the crosstalk between ROS and ncRNAs will facilitate the development of new ROS-based ncRNA-targeted or ncRNA medicine. Herein, we systematically review the progress of ncRNAs and ROS interacting with each other in cancer mainly from a therapeutic perspective, emphasizing the potential of ncRNA-based agents in clinical application.

## The production and therapeutic potential of ROS in cancer

ROS is primarily derived from mitochondria, whose oxidative metabolism efficiently produces ATPs to meet the energy demand of normal cells in the presence of oxygen. As a result of oxidative metabolism, the reactive species byproducts are generated, such as ROS. However, when mitochondrial dysregulation occurs, high ROS levels will release a ROS burst, causing mitochondrial destruction and even damaging the whole cell [39]. Generally, the antioxidant system can neutralize excessive ROS to prevent oxidative damage. While in cancer cells, elevated levels of ROS are commonly found, hence, it is confusing why cancer cells produce extra ROS and how they easily escape ROS damage and keep their malignant features of rapid proliferation, migration, invasion and apoptotic inhibition.

Metabolic stress, persistently occurs in tumor microenvironments, which implies a lack of nutrients, oxygen and growth factors because of excessive consumption via continuous proliferation and relatively insufficient angiogenesis of cancer cells [40], is a cause of ROS overload. Glucose deprivation of cancer cells directly leads to glycolysis blockade and ATP synthesis reduction, resulting in glycolysis-relevant antioxidant deficiency and provoking oxidative metabolism reoccurrence. Under such conditions, ROS production accelerates and elimination decreases, directly contributing to ROS accumulation.

Nevertheless, ROS levels within cancer cells can be modulated to a proper degree through their robust antioxidant systems to utilize oncogenic roles. Cancer cells exhibit a powerful antioxidant system consisting of reductants such as the antioxidant enzymes-superoxide dismutase (SOD), catalase (CAT), glutathione peroxidase (GPX) and the antioxidant agents-nicotinamide adenine dinucleotide phosphate (NADPH) and glutathione (GSH). Antioxidants sustain a moderate ROS level to protect cells from ROS attack and facilitate cancer progression. Antioxidant production is regulated by multiple signaling pathways and genes, such as nuclear factor erythroid 2-related factor 2 (Nrf2) [41]. Moderate ROS levels can decrease the toxicity of natural killer cells (NK cells) [42] and increase the self-renewal capacity of cancer cells. In addition, it can also activate multiple pro-tumorigenic signaling pathways such as NF-κB, TGF-β, JAK2-STAT1 and PI3K/Akt/ERK or activate oncogenes and inhibit tumor suppressors to enhance cancer progression [43–47]. Accordingly, through above intracellular self-adaptation alterations, ROS will be partially counteracted and cancer cells survive even can be further promoted.

**Fig. 1:**
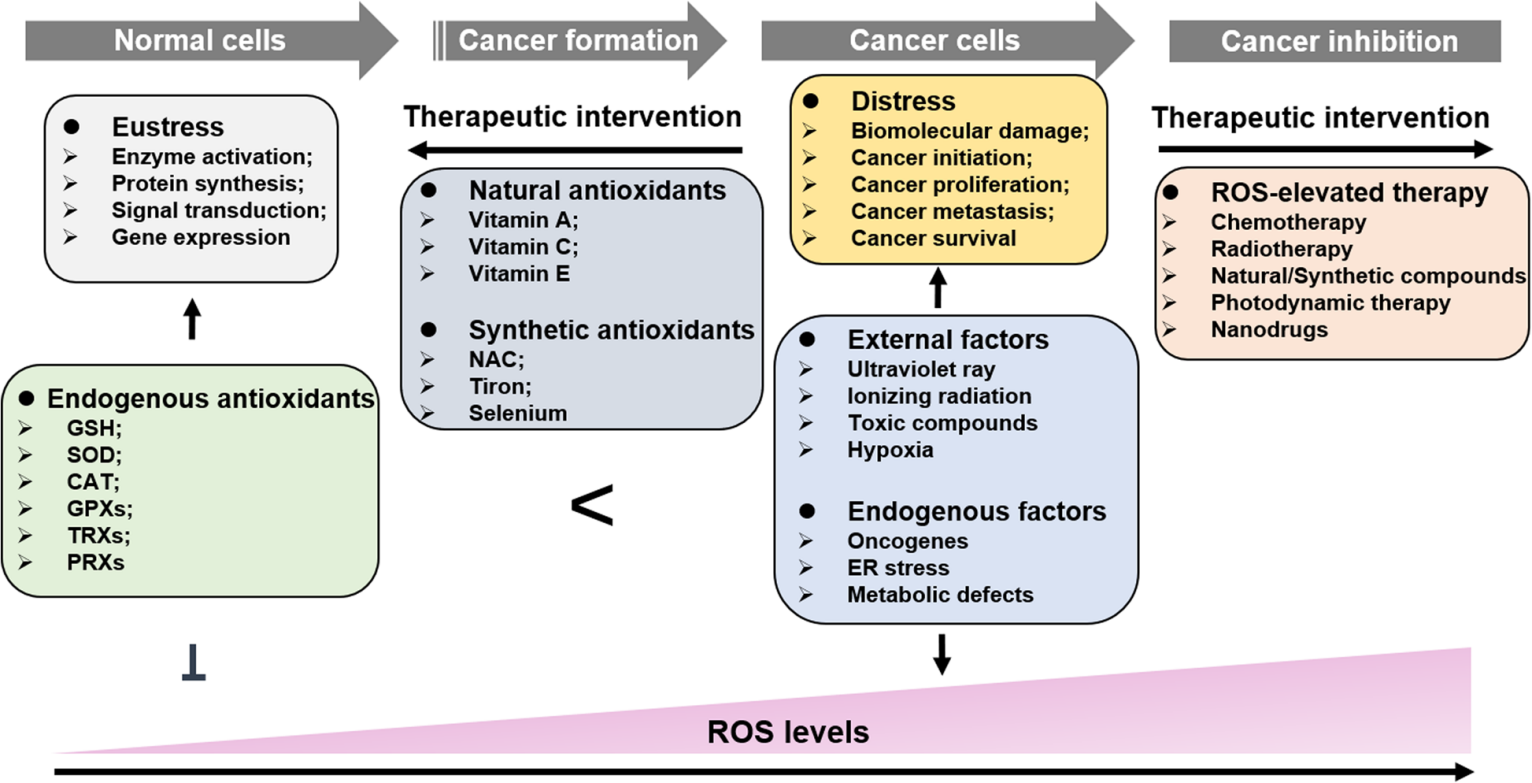
The production and therapeutic potential of ROS in cancer. In normal cells, the endogenous antioxidant system is enough to counteract excessive ROS to maintain redox homeostasis which benefits enzyme activation, protein synthesis, signal transduction and gene expression. Nevertheless, when the redox balance is broken, accumulated ROS will damage biological molecules and render malignant transformation. Notably, further elevated ROS levels can prevent cancer development even cause cancer cell death. The dual roles of ROS in cancer development remind us of two opposite treatments - antioxidant and pro-oxidant therapy

There should be a threshold value for ROS levels in regulating cell fate – malignant transformation and death, which may suggest underlying treatment regimens (Fig. 1). Normal cells, cancer cells and cancer stem cells (CSCs) have different intracellular ROS loads that may be the basis of ROS-elevated cancer therapy [48]. A higher level of ROS prompts a lower dose demand of ROS-elevated drugs/radiation in killing cancer cells thus, this is a complicated and significant course to explore appropriate therapeutic doses and ensure treatment benefits. While in most cases, patients who accept traditional chemo/radiotherapy show different side effects, including infections, anemia, fever, gastrointestinal discomfort and hair loss [49]. These impacts are probably derived from excessive treatments that cause ROS levels in normal cells to reach a toxic threshold and damage normal tissues. Moreover, decreasing treatment doses has difficulty killing most cancer cells and leads to cancer relapse. Additionally, CSCs have been universally acknowledged to be a pivotal element of cancer relapse. Stemness is a tough challenge for cancer therapy due to a lower ROS level in CSCs than that in normal cells.

In conclusion, although it shows great potential in cancer therapy, severe side effects are equally harmful. Hence, other replaceable therapeutic strategies on the foundation of ROS are urgently developed. Notably, ROS-based ncRNA-targeted or ncRNA medicine have exhibited some impressive achievements in cancer therapy, thus deeply understanding the crosstalk between ROS and ncRNAs will greatly promote the development of ROS-based agents and benefit cancer patients.

## The mechanisms of ROS-ncRNAs axis in cancer progression

Oxidative stress, that induces oxidative damage of biomacromolecules and aberrant modulation of redox signaling, is a key biological event throughout cancer initiation and development [50–52]. ROS as a second messenger during the moderate OS supports dysregulation of various pro-tumorigenic signaling pathways for malignant disease, in contrast, induction of excessive OS leads to cancer cell death by triggering ROS-driven apoptosis and ferroptosis [53, 54], thus indicating a potential development of OS-dependent strategy in clinical treatment. Accordingly, understanding the exquisite mechanism of redox modulation patterns facilitates a better therapeutic purpose by breaking redox homeostasis in cancer cells. NcRNAs occupy a majority of all transcripts, and are defined as dark matters in gene transcription at first. With in-depth investigation on the role of ncRNAs, they are commonly identified as being significantly involved in modulating carcinoma development [55–58], therefore displaying a promising potential as biomarkers or targets in cancer diagnosis and therapy [34, 35, 59, 60]. In this section, focusing on the detailed mechanism of the crosstalk between ncRNAs and ROS, we summarize the progress in recent years of ncRNAs involved in cancer initiation, metastasis and chemoradiotherapy resistance based on redox signaling regulation.

### Cancer initiation

Cancer initiation, featuring uncontrollable cell proliferation and growth, is the best stage for cancer prevention. Carcinogenic environmental factors with ROS induction properties have been proved to be one of the main causes in many cancer patients [61, 62]. A growing body of studies has revealed the underlying mechanisms associated with ncRNAs-ROS axis-driven cancer initiation. For example, hexavalent chromium and arsenic were reported to increase ROS-dependent *miR-21* transcription and compromise the expression of programmed cell death 4 (PDCD4), thus inducing the upregulation of downstream genes like E-cad, c-myc and uPAR and subsequently malignant transformation of bronchial epithelial cells [16, 63]. Furthermore, a previous study indicated that ionizing radiation (IR)-induced malignant transformation resulted in excessive OS by miR-21-mediated downregulation of the antioxidant enzyme superoxide dismutase 2 (SOD2) [17]. In line with this, UVB-triggered cancer initiation was revealed to hold a close relationship with hexavalent chromium and arsenic-derived OS based on modulation of miR-21 [18]. In addition, bronchial epithelial cells under chronic arsenic exposure were found to have accelerated cancerization, which was attributed to high ROS levels promoted the expression of hypoxia-inducible factor-1α (HIF-1α) and cyclooxygenases-2 (COX-2) by mitigating *miR-199a-5p* transcription [64].

In addition to miRNAs, lncRNAs involved in redox regulation also function in cancer initiation. The c-myc-downregulated lncRNA transcript IDH1-AS1 rendered the homodimerization of IDH1 and enhanced its enzymatic activity, which directly caused α-KG accumulation and OS and glycolysis blockage, preventing cancer proliferation at an early stage [65]. ROS-dependent lncRNA AX800134 was upregulated by TNF-α in hepatocellular carcinoma (HCC), which was elucidated to be responsible for the growth of cancer cells [66]. MACC1-AS1, an antisense of MACC1, was found to be increased in gastric cancer (GC). MACC1-AS1 maintained redox balance by stabilizing MACC1 mRNA and enhancing metabolic plasticity to promote the proliferation of cancer cells, which was indicated to be involved in AMPK/Lin28 signaling [67]. In melanoma, Wei et al found that lncRNA growth arrest-specific transcript 5 (GAS5) was significantly downregulated. The reduced transcription of *GAS5* greatly alleviated oxidative stress and accelerated cell cycle progression through EZH2/CDKN1C axis [68].

**Fig. 2:**
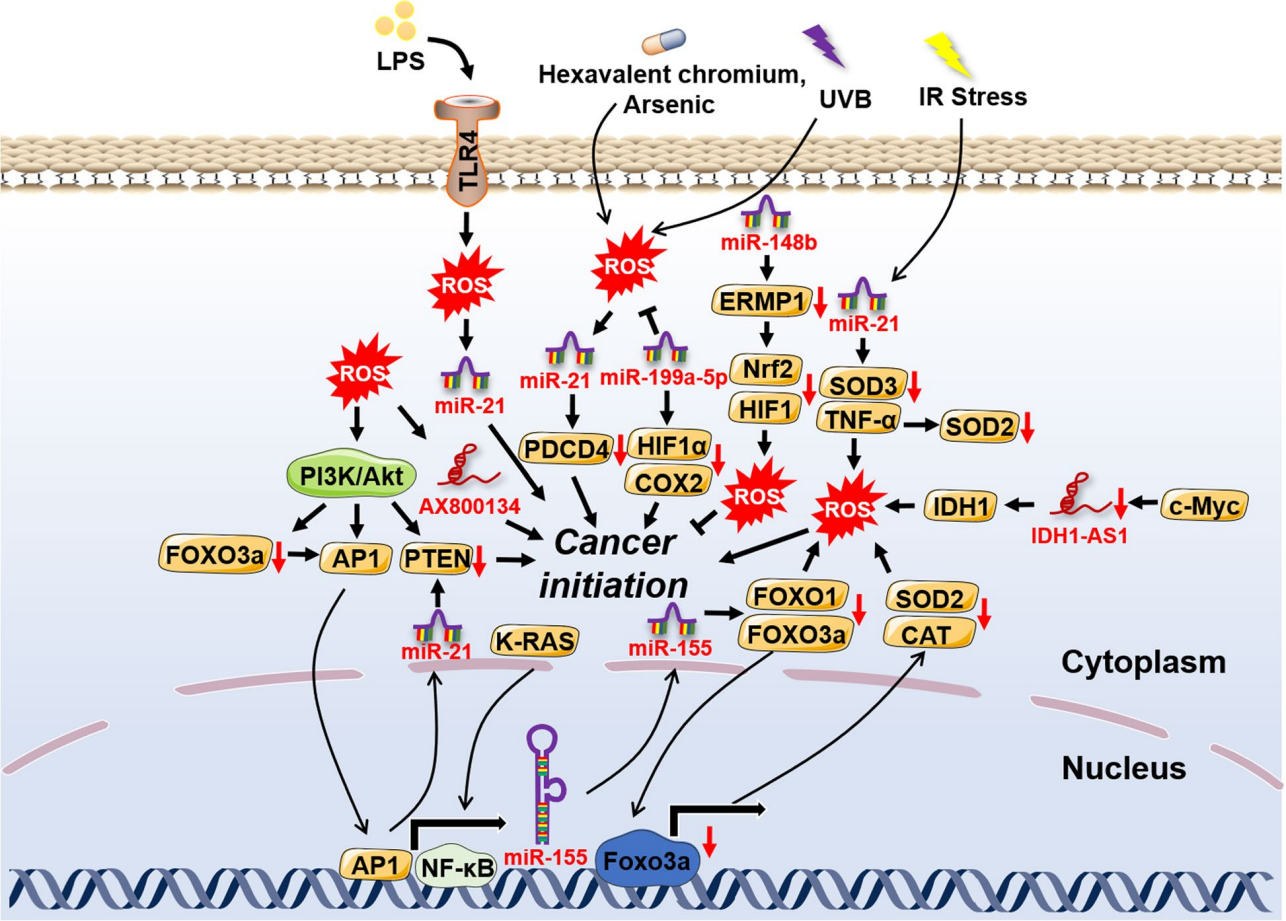
The crosstalk of ROS with ncRNAs in cancer initiation. The external factors, such as ultraviolet ray, ionizing radiation and toxic compounds, and internal factors caused by the aberrant gene regulation can both lead to cancer initiation through the complex interplay of ROS with ncRNAs

Although miRNAs and lncRNAs dominate in OS-mediated cancer initiation based upon available reports (Fig. 2), other ncRNAs still remain a large potential in the process. Although few studies have reported their interaction with ROS in cancer initiation, circRNAs have also been largely found to interact with ROS in other diseases and exhibit a promising role in cancer initiation. For instance, circRNA-101,036, identified as a tumor suppressor, induced ER stress and redox imbalance in oral squamous cell carcinoma [38]. As a sponge of miR-330-5p, tumor-suppressive human circular RNA (CircITCH) significantly reduced OS and alleviated doxorubicin-induced cardiotoxicity via elevating the expression levels of SIRT6, Survivin and SERCA2a [69]. Likewise, in intestinal ischemia/reperfusion, circ-PRKCB was reported to sponge endogenous miR-339-5p, modulating p66Shc expression and redox signaling [70].

### Cancer metastasis

**Fig. 3:**
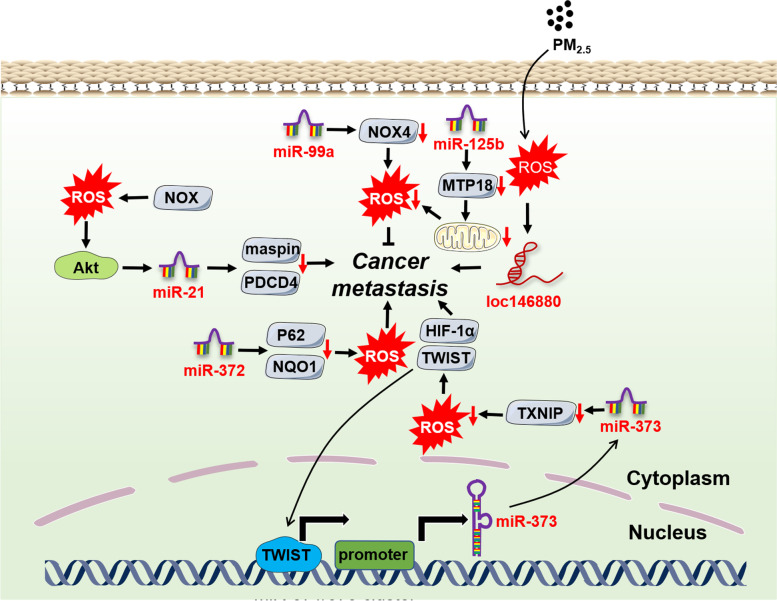
The crosstalk of ROS with ncRNAs in cancer metastasis. Cancer metastasis is tightly regulated by the intricate interaction of ROS with ncRNAs which involved multiple pivotal molecules and signaling pathways

Metastasis is one of the biggest obstacles in the treatment of terminal cancer patients. Understanding the detailed mechanisms in cancer metastasis may benefit the discovery of valuable therapeutic targets and the development of efficient interventions. Similarly, along with ROS, multiple types of ncRNAs have been confirmed to participate in cancer metastasis judging from current evidence (Fig. 3).

Epithelial-mesenchymal transition (EMT), characterized by the transition from epithelial cells to cells with a mesenchymal (M) phenotype, is a prerequisite of the metastasis for most solid cancers. EMT is triggered by some EMT-activating transcription factors (EMT-TFs), mainly of the SNAIL, TWIST and ZEB families and their targets like metalloproteinase-9 (MMP-9) [71]. During breast cancer progression, miR-373 quenched ROS levels by downregulating the ROS inducer thioredoxin-interacting protein (TXNIP), stimulating HIF-1α and TWIST expression [72]. Notably, TWIST bound to the promoter of the miR-371/373 cluster, which remarkably enhanced *miR-373* transcription. Hence, the miR-373-TXNIP-ROS-HIF1α-TWIST positive feedback signaling axis showed a powerful EMT-activating capacity. MiR-125b was found to be downregulated in HCC tissues, which prompted mitochondrial protein 18 kDa (MTP18) expression, leading to mitochondrial fission. Mitochondrial damage caused much ROS accumulation and increased MMP-9 expression, directly promoting EMT and cancer metastasis [73]. MiR-206 was reported to be modulated by nuclear factor NRF2 to drive tumorigenesis [74]. Simultaneously, miR-206 blocked TGF-β signaling and downstream neuropilin-1 (NRP1) and smad2 expression, inhibiting EMT, migration and invasion of breast cancer cells [75]. Oncogenic miR-21 level was reduced via kallistatin protein-mediated ROS downregulation, which further suppressed Akt signaling and EMT in cancer cells [76].

In addition to miRNAs, mounting evidence indicates that lncRNAs are also engaged in cancer metastasis. LncRNA UCA1 worked as a ceRNA, which prevented the effects of miR-1 and miR-203a on Slug expression, promoting EMT and invasion in breast cancer [77]. Similarly, lncRNA-MUF (mesenchymal stem cell-upregulated factor) can also act as a ceRNA of miR-34a, activating Snail1 expression and Wnt/β-catenin signaling to facilitate HCC metastasis [78]. Lnc-SNHG1 sponged miR-302/372/373/520 to enhance the expression of target genes-TGF-β receptor 2 (TGFBR2) and RAB11A, thus contributing to EMT in pituitary cancer [79]. Although the underlying mechanism remains unclear, we speculate that it is related to miR-1/34a/372-triggered ROS regulation from available reports [74, 80, 81]. LncRNA MALAT1 was found to upregulate ROS levels via keap1/Nrf1/2 signaling [82], which induced EMT and metastasis in head and neck squamous cell carcinoma (HNSCC) through STAT3 activation [83]. PM2.5 exposure of lung cancer cells significantly increased ROS levels and upregulated subsequent lncRNA loc146880 expression, causing malignant metastasis of lung cancer cells [84].

Besides miRNAs and LncRNAs, current studies have implied circRNAs are also capable of participating in OS-mediated cancer metastasis. CircSCAF11 overexpression inhibited ROS production by acting as a sponge of miR-145-5p in glioma, which is a cause of glioma initiation and metastasis [85]. MiR-30c-5p was reported to be an important OS regulator by multiple studies [86–88]. In colorectal cancer, circ3823 functioned as a ceRNA of miR-30c-5p, alleviating the repressive effect of miR-30c-5p on TCF7 which upregulated MYC and CCND1 and eventually led to cancer initiation and metastasis [89]. Hu et al identified an oncogenic circASAP1 through circRNA sequencing in patients with HCC, which was proved to be associated with cancer initiation and metastasis via miR-326/miR-532-5p-MAPK1/CSF-1 signaling, respectively [90]. The result suggested that the underlying mechanism was also modulated by OS [91, 92].

### Cancer chemoradiotherapy resistance

Chemoradiotherapy resistance is the leading threat for cancer patients. Reversing chemoradiotherapy resistance is a pivotal bottleneck problem to be resolved. OS-induced chemoradiotherapy resistance has been commonly identified, targeting which will be conducive to overcoming multidrug resistance (MDR) and radiotherapy failure [93].

Increasing evidence indicates that CSCs are at top and responsible for cancer chemoradiotherapy resistance, relapse and treatment failure [94]. It was reported that CSCs possessed a lower ROS level, which may be exploited to develop ROS-elevated treatments to reserve CSC-mediated chemoradiotherapy resistance. As a target of miR-223, the inhibition of HAX-1 led to mitochondrial damage and accelerated ROS generation in triple-negative breast cancer stem cells (TNBCSCs), which ultimately enhanced TRAIL-induced apoptosis [95]. MiR-153 downregulated Nrf2 and GPX1 expression, by which the elevated ROS levels decreased the stemness of glioma stem cells (GSCs) and relieved radiation resistance [96]. However, Yang et al reported that the decreased ROS levels resulting from miR-210 were able to weaken the stemness of hypoxic GSCs and reverse radiation resistance [97], which implied that CSC-induced chemoradiotherapy resistance should be further considered before applying redox agents. LncRNAs were also reported to participate in the process. For example, lncRNA H19 was upregulated in HCC tissues, knockdown of which caused OS and reversed the chemotherapy resistance of CD133+ CSCs via MAPK/ERK signaling [98].

**Fig. 4:**
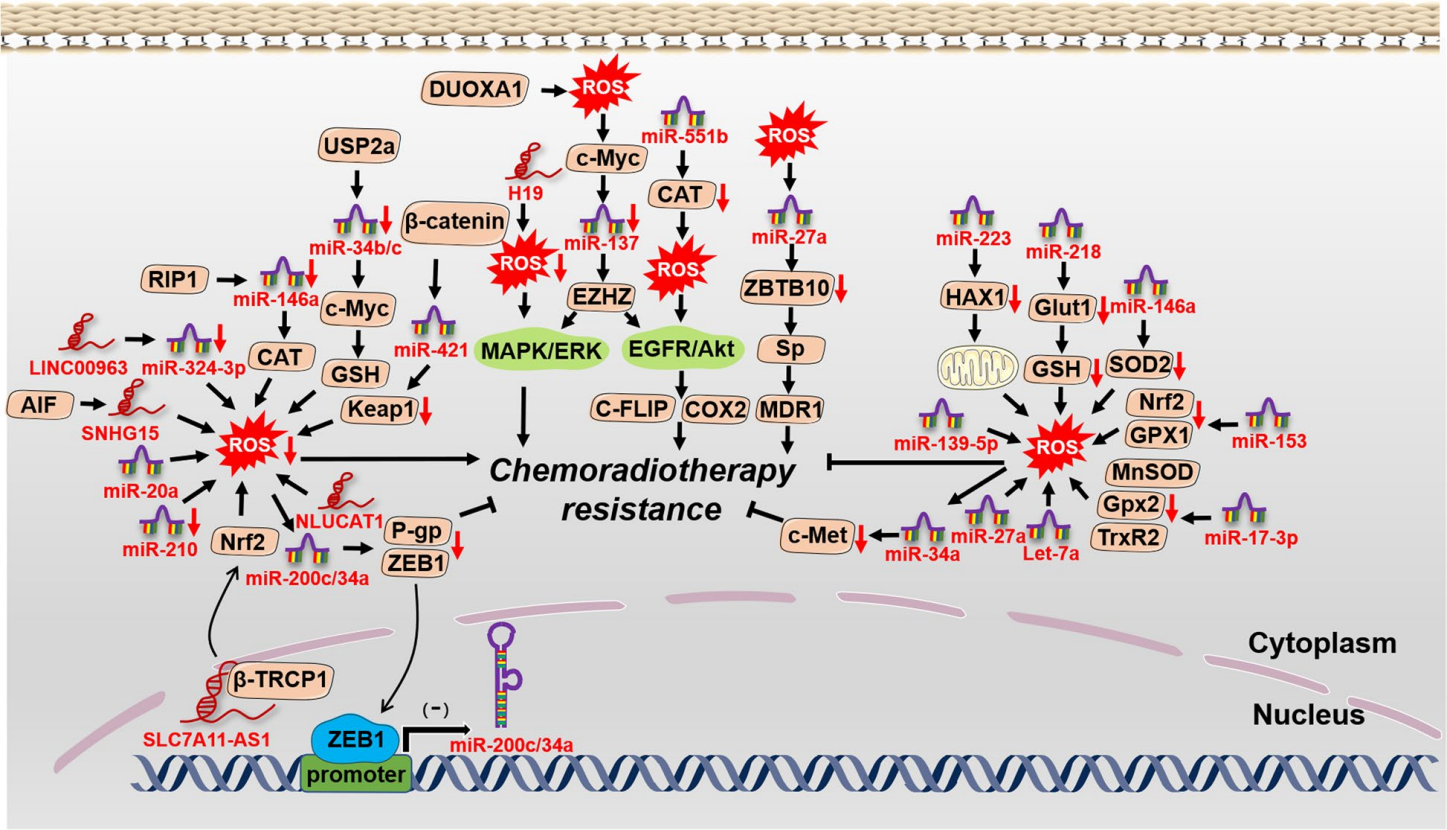
The crosstalk of ROS with ncRNAs in cancer chemoradiotherapy resistance. ROS interacts with ncRNAs, influencing the effects of chemoradiotherapy. The elaborate regulatory network provides us some possible therapeutic intervention targets in chemoradiotherapy resistance

Independent of CSCs, we find that the ncRNA-ROS axis can also influence chemoradiotherapy resistance through other biological processes on the basis of available evidence (Fig. 4). In breast cancer and melanoma, let-7a increased mitochondrial ROS production and improved the toxic effects of doxorubicin on cancer cells, although the underlying mechanism remained unclear [99]. MiR-17-3p elevated ROS levels by inhibiting three primary mitochondrial antioxidants, MnSOD, glutathione peroxidase 2 (Gpx2) and thioredoxin reductase 2 (TrxR2), remarkably strengthening the sensitivity of prostate cancer cells to IR [100]. MiR-34b/c prevented ubiquitin-specific protease 2a (USP2a)-c-myc-GSH signaling in prostate cancer, reversing cisplatin and doxorubicin resistance through ROS-induced apoptosis [101]. The promoters of *miR-200c-3p* and *miR-34a-3p* were occupied by zinc finger E-box binding homeobox 1 (ZEB1), which inactivated their transcription. Intriguingly, ROS-induced *miR-200c/34a* transcription targeted ZEB1 in turn, which greatly upregulated miR-200c/34a levels, inhibiting P-glycoprotein (P-gp) expression and reversing MDR [102]. Simultaneously, ROS-induced *miR-34a* transcription prevented c-Met expression and enhanced the cisplatin sensitivity of HCC cells [103].

LncRNAs were also found to play a pivotal part in enhancing the sensitivity of chemoradiotherapy in multiple studies. LncRNA NLUCAT1, identified from the hypoxic status of lung adenocarcinoma cell lines, facilitated proliferation and invasion via decreasing ROS levels with lower sensitivity of lung cancer cells to cisplatin-induced apoptosis [104]. LINC00963 was found to be elevated in several cancer types and associated with breast cancer invasion. Through sponging miR-324-3p, LINC00963 greatly relieved DNA damage and OS, rendering cancer progression and radiotherapy resistance [105]. LncRNA SLC7A11-AS1 was identified in gemcitabine-resistant pancreatic ductal adenocarcinoma (PDAC) cells, which bound with β-TRCP1 and prevented the ubiquitination and degradation of Nrf2 in the nucleus. A lower level of ROS mediated by Nrf2 increased the stemness of cancer cells, which benefited drug resistance [106].

Several reports have exhibited other ncRNAs were also involved in OS-modulated chemoradiotherapy resistance. CircCCND1 was remarkably upregulated in lung cancer cells and patients, which endowed cisplatin resistance of lung cancer cells by sponging miR-187-30 to modulate OS [107]. MiR-23b-3p has been proved to be closely related to chemotherapy resistance in multiple studies [108–111]. It suggested that circZNF292, acting as a ceRNA of miR-23b-3p to regulate anti-oxidative genes, may be a potential target in reversing chemotherapy resistance [112].

## Therapeutic implications based on the ROS-ncRNAs axis in cancer

Considering the great power of ROS and ncRNAs in regulating tumorigenesis and cancer progression, multiple preclinical studies targeting the ncRNAs-ROS axis have been conducted and achieved impressing treatment outcomes. ROS inducers and ncRNA-targeted or ncRNA agents are two primary druggable patterns with ncRNAs serving as both redox sensors and regulators, implying the large potential of modulating the ncRNAs-OS signaling axis in cancer therapy.

### ROS inducers in cancer therapy

#### Natural/synthetic compounds

Butyrate was found to be a ROS inducer through upregulating miR-22 expression and provoking mitochondrial dysfunction in HCC, directly causing cytochrome c release and caspase-3-dependent apoptosis [113]. Sulindac sulfide-triggered ROS decreased miR-27a expression, which prevented colon cancer cell growth via miR-27a/ZBTB10/specificity protein (Sp) axis. A similar process also occurred in Ethyl 2-((2,3-bis (nitrooxy) propyl) disulfanyl) benzoate (GT-094)-treated colorectal cancer [114, 115]. Phenethylisothiocyanate (PEITC) stimulated ROS generation, downregulating miR-27a/miR-20a/miR-17 expression. The process induced miRNA-modulated ZBTB10/ZBTB4/ZBTB34 expression, inhibiting Sp transcription factors-Sp1, Sp3, and Sp4 in pancreatic cancer cells. ROS-mediated Sp inhibition facilitated the apoptotic process, which may be a general mechanism of ROS-elevated cancer therapy [116]. However, quinacrine acted as a ROS inducer to upregulate FOXP3 by activating p38 MAPK and inactivating ERK. FOXP3-mediated miR-183 decreased β-TrCP mRNA stability, protecting Sp1 from degradation which enhanced pro-apoptotic protein Bax expression and the apoptosis of leukemia cells [117]. Besides, curcumin, betulinic acid (BA) and polygonatum odoratum lectin (POL) can also exert their antitumor effects via miRNA-mediated ROS accumulation [118–120].

#### Nanomaterials

With the development of nanotechnology, nano-delivery systems are promising in cancer diagnosis and therapy with lower toxicity and fewer side effects to normal cells. Among which the OS-mediated nanodrugs occupy a majority in cancer treatment. Mounting evidence has confirmed their effectiveness in preclinical studies. For example, in tumor acidy microenvironment, FeS@BSA nanocluster was degraded and produced much H2S and Fe2+, which synergistically released a ROS burst and evoked apoptosis of cancer cells [121]. Mesoporous silica nanoparticles (MSNs), featured with biochemical similarity with cell membranes, can both achieve anticancer and anti-angiogenesis purposes based on ROS [122]. Likewise, ZnO/CNT@FeO [123] and SASP/ZnO nanoparticles [124] can also increase ROS levels and facilitate cancer cell death. Though these studies are less reported about the detailed mechanisms, we hypothesis they must be closely related to the ROS-ncRNAs axis from above description.

### ncRNA-based agents in cancer therapy

A plethora of annotated ROS-relevant ncRNAs have been defined as therapeutic agents or targets in cancer, primarily including miRNAs, lncRNAs and circRNAs. Among which miRNAs are the most intensively investigated due to their dual roles and simplex functional patterns in cancer biology. Thus, deleting oncogenic miRNAs with anti-miRNA oligonucleotides (AMOs) and enhancing endogenous tumor suppressor miRNAs with miRNA mimics have become two main therapeutic strategies in preclinical and clinical studies. However, lncRNAs and circRNAs act through diverse functional repertoires in cancer, targeting which will require more basic research to avoid toxic effects. Nonetheless, several reports indicate that targeting oncogenic lncRNAs/circRNAs with double-stranded RNA-mediated interference (RNAi) and single-stranded antisense oligonucleotides (ASOs) significantly benefits cancer therapy, suggesting ncRNAs occupy a huge potential in developing cancer drugs.

#### MiRNAs

MiR-1293 was identified as a candidate for miRNA-based cancer therapeutics, mimicking which would alleviate OS by targeting BRD4 and significantly prevent the growth of colorectal cells in vitro and in vivo [125, 126]. MiR-34a was largely reported to be a tumor suppressor via silencing multiple OS-related oncogenes and signaling pathways, including SIRT1, IGF2BP3, FOXM1/eEF2K and IL-6R/STAT3 signaling axis, regulated cancer drug resistance, growth and metastasis [127–130]. Therefore, miR-34a upregulation with mimics or drugs has been widely applied in preclinical trials and shown impressive effects [131, 132]. For oncogenic miRNAs, AMO may be an option for cancer therapy. OS-mediated miR-21 was elucidated to be a pivotal oncogenic factor, that greatly facilitated cancer initiation, metastasis and drug resistance [63, 76, 97]. Yin et al exploited RNA micelles to precisely deliver the anti-miR-21 oligonucleotide into cancer cells, preventing the tumor-promoting function of miR-21 and engendering cell apoptosis in vitro and in vivo [133]. Likewise, Shu et al. developed a 15 nm therapeutic RNA nanoparticle to carry anti-miR-21, avoiding rapid physiological clearance and efficiently inhibiting the growth of triple negative breast cancer (TNBC) [134]. Besides miR-21, hypoxia-inducible miR-210 was also reported to prompt cancer progression in a hypoxic microenvironment [135, 136]. Combination polymeric CXCR4 antagonist (PCX)/anti-miR-210 nanoparticles was found to elicit cancer cell death and reverse drug resistance [137].

#### LncRNAs

LncRNAs act as both oncogenes and tumor suppressors in cancer biology. Despite their capacity in preventing cancer progression, few preclinical studies report specific “lncRNA mimics” function in carcinogenesis. ASOs and RNAi are two major strategies to target and inhibit oncogenic lncRNAs and improve cancer therapy. LncRNA MALAT1 was found to elevate ROS levels and facilitate HNSCC metastasis [83]. Gong et al constructed MALAT1-specific ASO Au nanoparticles with nucleus-targeting TAT peptide, successfully delivering ASO into lung cancer cells and reducing metastatic tumor nodules in vivo [138]. Vascular endothelial growth factor A (VEGFA) was proved to exacerbate OS and contribute to cancer progression [139]. LINC00173.v1, another oncogenic lncRNA, was discovered to promote the angiogenesis and development of lung cancer [140]. The specific ASO against LINC00173.v1 was investigated in vivo and it revealed a better anticancer outcome and enhanced sensitivity to cisplatin in lung cancer. SiRNA-mediated RNAi has been intensively applied to silence protein-coding genes, benefiting the prognosis of multiple diseases including cancer. Silencing oncogenic lncRNAs equally improves cancer treatment. FLANC, a novel primate-specific lncRNA, was identified as a potential therapeutic target based on clinical data [141]. Encapsulating FLANC siRNA in 1,2-dioleoyl-sn-glycero-3-phosphatidylcholine nanoparticles, Pichler et al confirmed si-FLANC dramatically decreased metastases without any side effects. Mechanistically, si-FLANC reduced STAT3-induced VEGFA expression, inhibiting OS and cancer progression. In addition, si-MALAT1 with a nanocarrier was also an optional approach for overcoming drug resistance, which substantially enhanced the sensitivity of glioblastoma to temozolomide (TMZ) [142].

#### CircRNAs

CircRNAs are a novel subtype of ncRNAs identified in recent years. Nonetheless, they have been revealed to be essential in cancer biology through interacting with redox sensors or regulators, thus a therapeutic role can also be represented on the basis of circRNAs. CircMMP9 was found to act as a sponge of the redox regulator miR-124, accelerating the growth and metastasis of glioblastoma multiforme (GBM) cells [143, 144]. The administration of circMMP9 siRNA significantly prevented cancer growth and metastasis in vitro and in vivo. Similarly, circAF4 functioned as an oncogene to sponge the other redox regulator-miR-128-3P, upregulating MLL-AF4 fusion protein expression and inducing leukemogenesis. Si-circAF4 treatment showed a remarkable cancer-preventing outcome through evoking leukemic cell apoptosis [145]. In addition to siRNAs, ASOs targeting oncogenic circRNAs also indicate the huge therapeutic potential in treating cancer. CircRNA activating MAFF (cia-MAF) was identified as a functional oncogenic circular RNA in liver cancer and liver tumor-initiating cells (TICs). The ASO specific to cia-MAF displayed impaired self-renewal and metastatic capacities of liver cancer via promoting the expression of redox sensor-MAFF [146, 147]. CircPGR was identified by circRNA sequencing (circRNA-seq) under estrogen inducement, which modulated estrogen receptor (ER)-positive breast cancer cell growth via sponging the redox regulator miR-301a-5p. CircPGR ASO administration significantly prevented the growth of ER-positive breast cancer [148, 149].

## Clinical applications of ROS-related ncRNAs in cancer patients: diagnostic value and therapeutic strategies

In view of the potent effects of OS-related ncRNAs on all major cancer hallmarks, a multitude of preclinical research have explored the rationality of targeting these ncRNAs as mentioned above. Current evidence indicates many ROS-related ncRNAs are rather promising in cancer diagnosis and therapy, some of which have been developed as therapeutic agents and targets. Small ncRNAs such as miRNAs and siRNAs have been widely applied to clinical trials in multiple diseases, whereas fewer lncRNA or circRNA-based therapeutics have entered the clinic [150]. In this section, we summarize the clinical advancements of ROS-related ncRNAs in cancer diagnosis and therapy, emphasizing more efforts should be made to test and develop more ncRNA-based drugs. Partial ROS-related ncRNAs that enter the clinic are listed in Table 1.

**Table 1:** Clinical applications of partial ROS-related ncRNAs in cancer patients

NcRNA	Type	Cancer	Application	Status	Identifier
MiR-373	NA	Breast cancer	Cancer diagnosis	Recruiting	NCT04720508
MiR-155	NA	Bladder cancer	Cancer diagnosis	Completed	NCT03591367
MiR-371	NA	Germ cell cancer	Cancer diagnosis	Recruiting	NCT04435756
HOTAIR	NA	Thyroid cancer	Cancer diagnosis	Unknown	NCT03469544
MiR-16	Mimic	Pleural mesothelioma lung cancer	Cancer therapy	Phase I	NCT02369198
MiR-122	ASO	Liver cancer	Cancer therapy	Phase I/II	NCT01646489, NCT01727934, NCT01872936, NCT01200420
MiR-155	ASO	T-cell lymphoma	Cancer therapy	Phase II	NCT03713320, NCT03713320
MiR-34a	Mimic	Melanoma	Cancer therapy	Phase I	NCT01829971, NCT02862145
MtlncRNA	ASO	Unresectable tumors	Cancer therapy	Phase I	NCT02508441, NCT03985072

### Cancer diagnosis

#### MiRNAs

MiR-373 functioned as a redox regulator and was found to promote tumorigenesis in multiple cancers [72, 151, 152]. In a clinical study concerning the diagnosis of breast cancer, miR-373 was exploited as a serum biomarker to evaluate its correlation with clinicopathological documented data, staging, grading and tumor receptors (NCT04720508). Similarly, some research reported that miR-155 was oncogenic in several cancers through targeting Nrf2-mediated OS [153–155], which was considered as a promising biomarker coupled with telomerase reverse transcriptase (TERT) in a clinical exploration through assessing the potential roles in the diagnosis of non-muscle-invasive bladder cancer and their correlation with stage and grade (NCT03591367). Another oncogenic redox regulator miR-371, was largely reported to cause the growth, metastasis and drug resistance of cancers [72, 156–158]. On the foundation of differentially expressed levels, Nichols et al carried out a clinical trial to estimate the positive predictive value of miR-371 in patients with recurrent germ cell cancers (NCT04435756). In pancreatic cancer, limited by the specificity of the FDA-approved biomarker CA199, small RNAs have been considered as promising biomarkers owing to their numbers and stability, miRNAs included. These small RNAs from circulating extracellular exosomes between pancreatic cancer patients and healthy controls are being analyzed to discover specific diagnostic biomarkers through next-generation sequencing (NCT04636788). Similarly, another clinical trial exploited urinary exosomes to discern and validate the predictive role of candidate exosomal microRNAs in aggressive prostate cancer (NCT03911999). Nonspecific to a certain miRNA (34 miRNAs were assessed), a study attempted to search for specific miRNA markers for earlier diagnosis of lung cancer by quantifying miRNAs from peripheral blood (NCT03293433).

#### LncRNAs and circRNAs

LncRNA and circRNA-based clinical research are less conducted except for the redox regulator-lncRNA HOTAIR (NCT03469544). Nonetheless, the whole lncRNA/circRNA sequencing to uncover novel biomarkers has been carried out. For instance, in neuroendocrine neoplasm (NEN), circular RNA sequencing was used to screen candidate circRNAs that potentially acted as diagnostic and predictive biomarkers, following subsequent validation between the neuroendocrine tumor group and control group (NCT04464122). Likewise, lncRNA sequencing can also benefit cancer diagnosis. Considering the availability and detectability of exosomes, two clinical trials tried to extract exosomal RNAs from the plasma of patients with lung cancer/high grade serous ovarian cancer (HGSOC) and healthy individuals, aiming to identify multiple specific lncRNA and lncRNA/miRNA biomarkers through next-generation RNA sequencing (NCT03830619, NCT03738319). In addition, RNA profiling through the whole RNA sequencing of clinical specimens may suggest some unexpected lncRNA/circRNA biomarkers in cancer diagnosis. In metastatic high-grade osteosarcoma, Shen et al collected blood samples between metastatic and non-metastatic osteosarcoma patients to extract exosomes and perform RNA profiling, aiming to identify specific RNA biomarkers for osteosarcoma metastasis through next-generation RNA sequencing (NCT03108677). We believe these explorations will conduce to observing more valuable OS-related lncRNA/circRNA biomarkers based on clinical specimens.

ROS-related ncRNAs not only serve as direct biomarkers to help distinguish cancer patients, but also facilitate clinicians to monitor redox homeostasis and evaluate the risk of suffering redox-related diseases. Thus, identifying more ROS-related ncRNA biomarkers is essential for diagnosing redox-related diseases including cancer at an early stage.

### Cancer therapy

#### MiRNAs

Though miRNA-mediated preclinical studies with significant anticancer effects have been extensively reported, relevant clinical trials are less carried out. MiR-16 was able to downregulate OS and inhibit the progression of multiple cancers, functioning as a tumor suppressor in malignant pleural mesothelioma, lung cancer breast cancer and leukemia [159–163]. In malignant pleural mesothelioma and non-small cell lung cancer, miR-16 mimic was packaged in minicells targeted with epidermal growth factor receptor (EGFR)-specific antibodies, which exhibited an acceptable safety profile in a phase I study [164] (NCT02369198). Some miRNA-based drugs may not be tested directly for cancers in clinical trials, but we speculate they do good to cancer prevention as well. For example, the hepatitis C virus (HCV) infection has been proved to be closely related to HCC, avoiding which is essential to HCC prevention. Miravirsen (SPC3649), a developed anti-miR-122 agent, was considered to efficiently alleviate HCV infection in multiple phase I/II investigations (NCT01646489, NCT01727934, NCT01872936, NCT01200420). However, some other miRNA-based clinical trials were terminated due to the limited safety or efficacy. Several findings uncovered miR-155 was capable of inducing OS and engendering cancer metastasis and chemoresistance via multiple genes and signaling pathways [165–167], targeting and inhibiting which may benefit cancer treatment. Two phase II investigations were designed to evaluate the efficacy and safety of cobomarsen (a miR-155 inhibitor) in patients with cutaneous T-cell lymphoma (CTCL) and mycosis fungoides (MF), accompanied by a comparation to vorinostat, a drug that has been approved for the treatment of CTCL (NCT03713320, NCT03713320). In addition, miR-34a was universally identified as a tumor suppressor through redox regulation, whose analogues were widely applied in animal models [78, 101, 102]. MRX34, a miR-34a mimic, was exploited as an intervention agent to test the safety, pharmacokinetics and pharmacodynamics in a phase I study (NCT01829971) and assess the efficacy of combined treatment with dexamethasone, a phase II drug for melanoma patients (NCT02862145). Unfortunately, these promising clinical studies were forced to end on account of the significant side effects and insufficient efficiency.

#### LncRNAs

Most investigations targeting conventional lncRNAs are still in the preclinical stage, though a multitude of inspiring results have suggested that lncRNA-based drugs are very promising. Even so, it is conceivable that the entrance of lncRNA-based therapeutics into clinical studies is imminent. Herein, we notice that mitochondrial lncRNAs (mtlncRNA), specifically transcribed by mitochondrial DNA, have obtained rapid advancements [168]. Antisense noncoding mitochondrial RNA (ASncmtRNA) was identified as a redox sensor, with an oncogenic role in the progression of various cancers [169–171]. These preliminary results implied the potential of ASncmtRNA-targeting ASOs in clinical cancer treatment. To this end, Andes-1537 was approved to treat multiple advanced unresectable solid tumors by targeting ASncmtRNA with a short single-stranded phosphorothioate ASO (NCT02508441, NCT03985072). The phase I study demonstrated that Andes-1537 is a well-tolerated drug [172].

#### Natural compounds targeting ROS-related ncRNAs

Additionally, though not claiming lncRNAs or circRNAs as direct targets, some natural compounds have entered the clinic and been exploited as anticancer agents. Curcumin was found to relieve OS through modulating Nrf2 expression, exerting antitumor activity and enhancing the sensitivity of chemoradiotherapy via lncRNA and circRNA regulation [173–178]. A clinical practice used for observing the roles of curcumin in intestinal adenomas showed neither significant adverse effects nor anticancer functions (NCT00927485). Dietary supplementation with curcumin to assess the radiosensitizing and radioprotective effects in prostate cancer was completed in a clinical trial, though the detailed results were not posted (NCT01917890). Another phase II clinical study tested the function and safety of curcumin in advanced pancreatic cancer, suggesting the huge potential of curcumin in clinical application [179] (NCT00094445). Similarly, resveratrol acted as a redox regulator, remarkably decreasing OS levels and inhibiting cancer development through activating sirtuin 1 (SIRT1) and lncRNA-modulated pathways [180, 181]. Clinical trials have been exploited to investigate the therapeutic outcomes of resveratrol in colorectal cancer (NCT00256334, NCT02261844) and liver cancer (NCT02261844), which exhibited an impressing tumor preventive role in cancer patients [182].

## Concluding remarks and future perspectives

OS is a double-edged sword. Oxidative eustress activates redox signaling and promotes growth and migration. Nevertheless, oxidative distress stimulates oxidative damage, growth inhibition and cell death. The dual roles remind us of two types of therapeutic methods targeting ROS. Though some antioxidant agents have been elucidated to efficiently prevent oxidative damage and cancer development, they may not always benefit or even be at risk. Most treatments are still to increase ROS levels that partially owe to breaking the redox equilibrium of cancer cells. Although the strategies have acquired gratifying achievements, disadvantages such as low sensitivity of cancer cells, low abundance within cancer cells and side effects on normal cells are equally significant. Therefore, ROS-based treatments should be revisited from a whole new perspective.

**Fig. 5:**
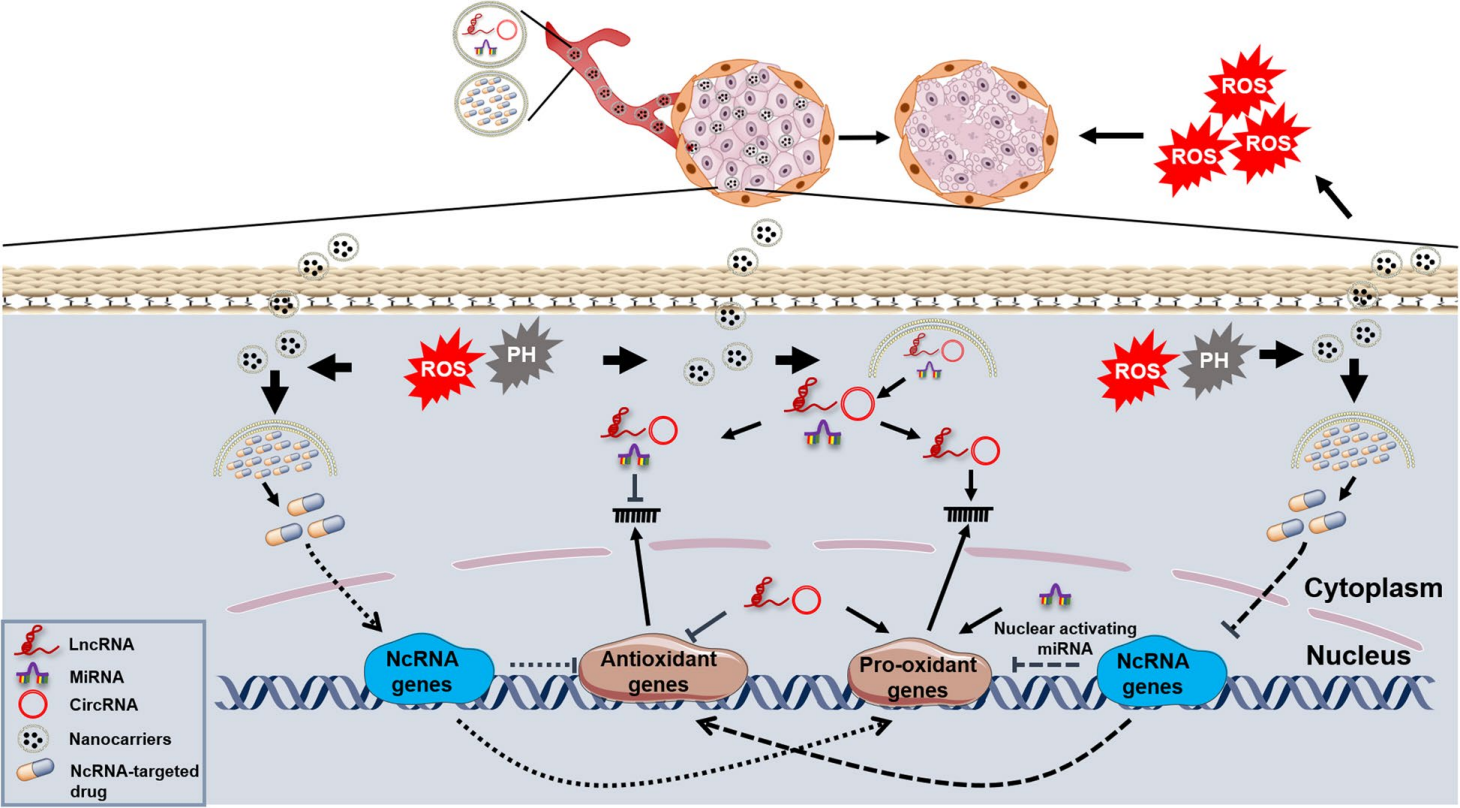
The prospects for developing ROS-based ncRNA and ncRNA-targeted nanodrugs. In view of the robust therapeutic capacity of elevating ROS levels and ncRNAs being crucial ROS regulators, developing ncRNA and ncRNA-targeted drugs may be optional strategies to cure cancer. Nanodrugs are endowed a targetable characteristic under the special conditions, for example, they can selectively release contents within cancer cells due to the acidic PH and high ROS levels. Thus, ncRNA and ncRNA-targeted nanodrugs can specifically regulate antioxidant and pro-oxidant genes to exacerbate redox imbalance, killing cancer cells through releasing a ROS burst

NcRNAs, especially for miRNAs and lncRNAs, can interact with ROS in cancer development and chemotherapy/radiotherapy resistance. Given the strong tissue and space-time specificity and conservative property of ncRNAs, directly developing ROS-based ncRNA and ncRNA-targeted drugs may cure the specific cancer types. In addition, the functional mechanisms of ncRNAs are simplex, making it probable that exploit highly targeted and low-toxicity ncRNA agents. With the rapid development of biotechnologies, more ncRNAs are being discovered, and their precise regulatory patterns will be identified. It is capable to establish a biobank, aiming to illuminate the ncRNA-ROS interaction network and promote the progression of ROS-based ncRNA or ncRNA-targeted medicine. Additionally, more advanced nanocarriers will make it promising that all different kinds of ncRNAs can be packaged and quickly enter cancer cells regardless of their length and biocompatibility (Fig. 5).

Notably, the tissue specificity of ncRNAs suggests that they may occupy significant parts in tissue formation, growth, differentiation, development, etc. The altered expression of ncRNAs with human intervention may be a risk on account of upregulating or downregulating ncRNAs can both cause tissue dysfunction and even damage. Aerobic glycolysis, which frequently occurs in multiple tumor tissues and determines the survival of cancer cells, is a crucial target for the ROS-ncRNA axis in cancer therapy. However, we can never only focus on the process to treat cancers. Mounting evidence indicates that in different cancer types and progressive stages, cancer cells exhibit unique metabolic forms. Furthermore, there are still many other challenges limiting the imagination. Most lncRNAs remain unexplored, which is a large obstacle for establishing a complete ncRNA-ROS interaction network to find more therapeutics. These are considerable for safe precision medicine, thereby the extensive basic research and long-term clinical observations must be carried out before relevant drugs being developed and entering the markets. Altogether, ROS-based ncRNA or ncRNA-targeted medicine holds promise in cancer therapy but still requires more investigations to ensure their safety and effectiveness.

## Data Availability

Not applicable.

## Abbreviations

OS: Oxidative stress
ROS: Reactive oxygen species
NcRNAs: Noncoding RNAs
RNS: Reactive nitrogen species
RSS: Reactive sulfur species
RCS: Reactive chlorine species
ER: Endoplasmic reticulum
MiRNAs: MicroRNAs
CircRNAs: Circular RNAs
SiRNAs: Small interfering RNAs
SnRNAs: Small nuclear RNAs
SnoRNAs: Small nucleolar RNAs
PiRNAs: PIWI-interacting RNAs
tsRNAs: tRNA-derived small RNAs
eRNAs: enhancer noncoding RNAs
SOD: Superoxide dismutase
CAT: Catalase
GPX: Glutathione peroxidase
NADPH: Nicotinamide adenine dinucleotide phosphate
GSH: Glutathione
Nrf2: Nuclear factor erythroid 2-related factor 2
CSCs: Cancer stem cells
PDCD4: Programmed cell death 4
IR: Ionizing radiation
HIF-1α: Hypoxia-inducible factor-1α
COX-2: Cyclooxygenases-2
HCC: Hepatocellular carcinoma
GC: Gastric cancer
GAS5: LncRNA growth arrest-specific transcript 5
EMT: Epithelial-mesenchymal transition
EMT-TFs: EMT-activating transcription factors
MMP-9: metalloproteases-9
TXNIP: Thioredoxin-interacting protein
MTP18: Mitochondrial protein 18 kDa
NRP1: Neuropilin-1
TGFBR2: TGF-β receptor 2
HNSCC: Head and neck squamous cell carcinoma
MDR: Multidrug resistance
TNBCSCs: Triple-negative breast cancer stem cells
GSCs: Glioma stem cells
Gpx2: Glutathione peroxidase 2
TrxR2: Thioredoxin reductase 2
USP2a: Ubiquitin-specific protease 2a
ZEB1: Zinc finger E-box binding homeobox 1
P-gp: P-glycoprotein
PDAC: Pancreatic ductal adenocarcinoma
Sp: Specificity protein
PEITC: Phenethylisothiocyanate
BA: Betulinic acid
POL: Polygonatum odoratum lectin
MSNs: Mesoporous silica nanoparticles
AMOs: Anti-miRNA oligonucleotides
RNAi: RNA-mediated interference
ASOs: Antisense oligonucleotides
TNBC: Triple negative breast cancer
PCX: polymeric CXCR4 antagonist
VEGFA: Vascular endothelial growth factor A
TMZ: Temozolomide
GBM: Glioblastoma multiforme
Cia-MAF: CircRNA activating MAFF
TICs: Tumor-initiating cells
CircRNA-seq: CircRNA sequencing
ER: Estrogen receptor
TERT: Telomerase reverse transcriptase
NEN: Neuroendocrine neoplasm
HGSOC: High grade serous ovarian cancer
EGFR: Epidermal growth factor receptor
HCV: Hepatitis C virus
CTCL: Cutaneous T-cell lymphoma
MF: Mycosis fungoides
MtlncRNA: Mitochondrial lncRNAs
ASncmtRNA: Antisense noncoding mitochondrial RNA
SIRT1: Sirtuin 1
